# Supplementary material for: Mapping cryptic phosphorylation sites in the human proteome
Source: EMBO J. 2025 Oct 3;44(22):6704–31. doi: 10.1038/s44318-025-00567-1 (PMC12624043; doi:10.1038/s44318-025-00567-1)
Supplement: Supplementary file 1 — Appendix [file 44318_2025_567_MOESM1_ESM.pdf]

Appendix for

Mapping Cryptic Phosphorylation Sites in the Human Proteome

Table of Contents

Item	Page
Appendix Table S1. Proportion of cryptic or non-cryptic phosphosites associated with disease-related mutations in each group from the COSMIC and PTMVar datasets.	2
Appendix Table S2. Cross-referenced cryptic phosphosites replaced by a phosphomimetic mutation in PTMvar.	3

Appendix Table S1. Proportion of cryptic or non-cryptic phosphosites associated with disease-related mutations in each group from the COSMIC and PTMVar datasets. The number of entries for each dataset, the total number of phosphosites and their frequency are reported for cryptic and non-cryptic phosphosites.

	N. Entries	Total n. Phosphosites	Frequency
	**COSMIC**		
**Non-Cryptic**	4055	207225	1.96%
**Cryptic**	204	10606	1.92%
	**PTMVar**		
**Non-cryptic**	1270	207225	0.61%
**Cryptic**	138	10606	1.30%

Appendix Table S2. Cross-referenced cryptic phosphosites replaced by a phosphomimetic mutation in PTMvar. The table is a subset of Table EV4. Entries are:

- **ROW_ID:** Index of the row
- **GENE:** Principal gene name, in most cases is the accepted HGNC symbol.
- **UPID:** UniProtKB ID for GENE.
- **FTID:** The UniProtKB/Swiss-Prot UID of the variant.
- **WT_AA:** Wild type amino acid.
- **VAR_AA:** Mutated amino acid.
- **VAR_TYPE:** LP/P = likely pathogenic or pathogenic, LB/B = likely benign or benign, US = uncertain significance.
- **DISEASE(s):** Specific disease(s) associated with the mutation. Includes the abbreviation and UID from the Online Mendelian Inheritance in Man® (OMIM®). For example, the abbreviation and UID for phenylketonuria are PKU and MIM:261600.
- **MUT_SOURCE:** The sources of mutant data include HUMSAVAR (www.uniprot.org/docs/humsavar), The Cancer Genome Atlas (TCGA; cancergenome.nih.gov/), Catalogue Of Somatic Mutations In Cancer (COSMIC; cancer.sanger.ac.uk/cancergenome/), and the cBioPortal for Cancer Genomics (cBIO; www.cbioportal.org/).
- **MOD_RSD:** Sequence number of the residue that is mutated.
- **VAR_CLASS:** CLASS I (site loss) [STY]→{STY} OR [KR]→{KR}, CLASS Ia (modsite switch) [Y]→[ST] OR [TS]→[Y], or CLASS II (flanking change) variant +/- 5 AAs from the modification site. We include only CLASS I variants.
- **ONC_TSG:** Role of the gene in cancer.

ROW_ID	GENE	UPID	FTID	WT_AA	MUT_RSD#	VAR_AA	VAR_TYPE	DISEASE(s)	MUT_SOURCE	MOD_RSD	VAR_CLASS	ONC_TSG
**1**	ANOS1	P23352	VAR_069207	Y	217	D	US	-	Uniprot Humsavar	217	I	
**2**	CYBB	P04839	VAR_025613	Y	41	D	LP/P	Granulomatous disease, chronic, X-linked (CGDX) [MIM:306400]	Uniprot Humsavar	41	I	
**3**	F8	P00451	VAR_028533	Y	450	D	LP/P	Hemophilia A (HEMA) [MIM:306700]	Uniprot Humsavar	450	I	
**4**	HBB	P68871	VAR_003063	Y	131	D	LB/B	-	Uniprot Humsavar	131	I	
**5**	HLA-DPB1	P04440	VAR_060642	Y	57	D	LB/B	-	Uniprot Humsavar	57	I	

**6**	IDS	P22304	VAR_007351	Y	225	D	LP/P	Mucopolysaccharidosis 2 (MPS2) [MIM:309900]	Uniprot Humsavar	225	I	
**7**	PLCG2	P16885	TCGA-13-1498-01A	Y	1036	D	Disease	ovarian_cancer[TCGA]	TCGA	1036	I	
**8**	TGM1	P22735	VAR_058673	Y	365	D	LP/P	Ichthyosis, congenital, autosomal recessive 1 (ARCI1) [MIM:242300]	Uniprot Humsavar	365	I	
**9**	TP53	P04637	VAR_045116	Y	220	D	US	Sporadic cancers	Uniprot Humsavar	220	I	oncogene, TSG, fusion
**10**	UHMK1	Q8TAS1	VAR_041273	Y	197	D	LB/B	-	Uniprot Humsavar	197	I	
**11**	VHL	P40337	VAR_005766	Y	175	D	LP/P	Von Hippel-Lindau disease (VHLD) [MIM:193300]	Uniprot Humsavar	175	I	TSG
