# Supplementary material for: Mapping cryptic phosphorylation sites in the human proteome
Source: EMBO J. 2025 Oct 3;44(22):6704–31. doi: 10.1038/s44318-025-00567-1 (PMC12624043; doi:10.1038/s44318-025-00567-1)
Supplement: Supplementary file 7 — Expanded View Figures [file 44318_2025_567_MOESM7_ESM.pdf]

Expanded View Figures

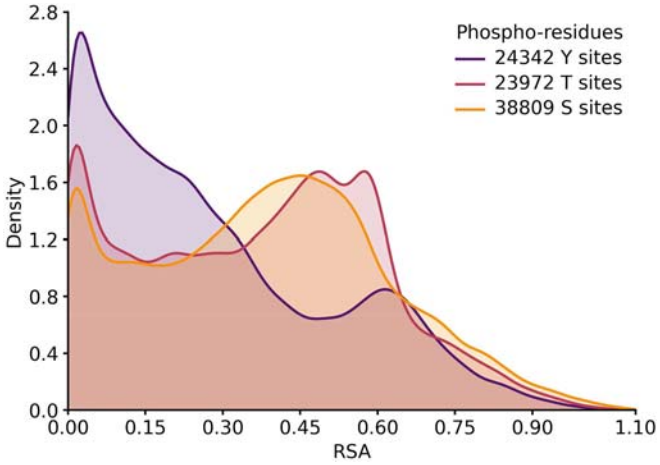

Figure EV1. Graphic distribution of the occurrence of phosphorylation in relation to the RSA of the corresponding target amino acids (serine, tyrosine and threonine).

© The Author(s)

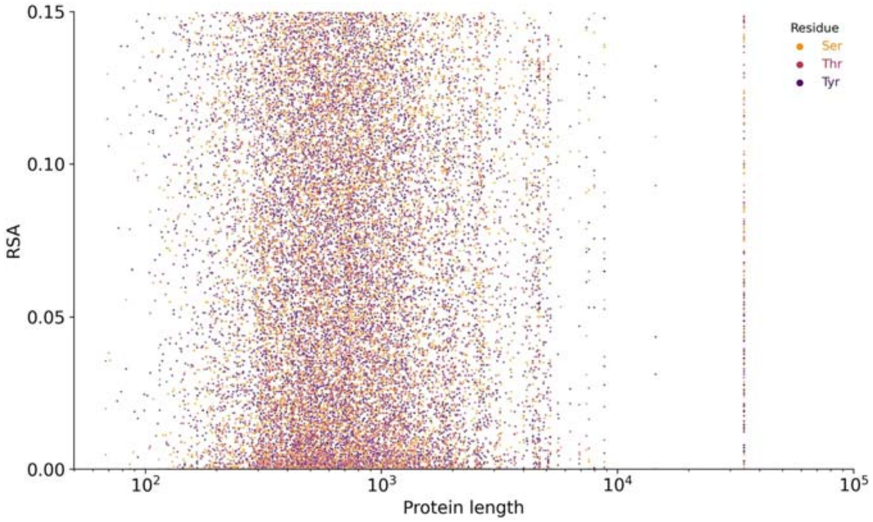

Figure EV2. Graphical plot of the RSA of cryptic phosphosites vs the length of the proteins in which they occur.

 © The Author(s)

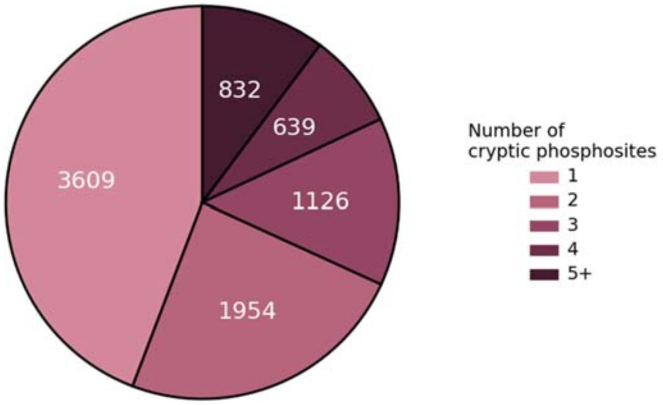

Figure EV3. Pie chart illustration of the number of cryptic phosphosites per protein.

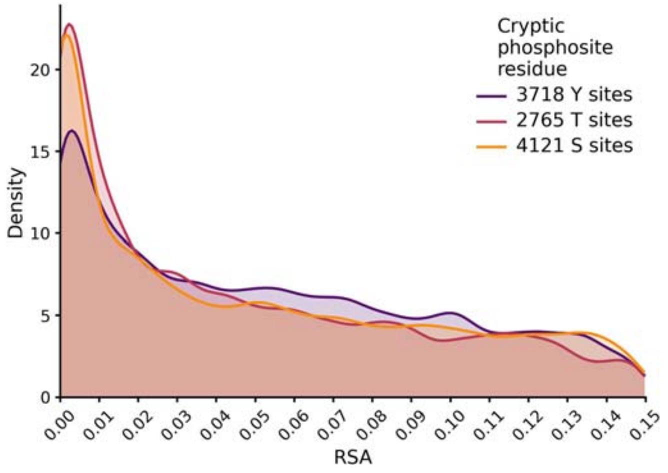

Figure EV4. Graphic distribution of the occurrence of phosphorylation in relation to the RSA of the corresponding amino acids after applying the dynamic and structural filtering.

 © The Author(s)

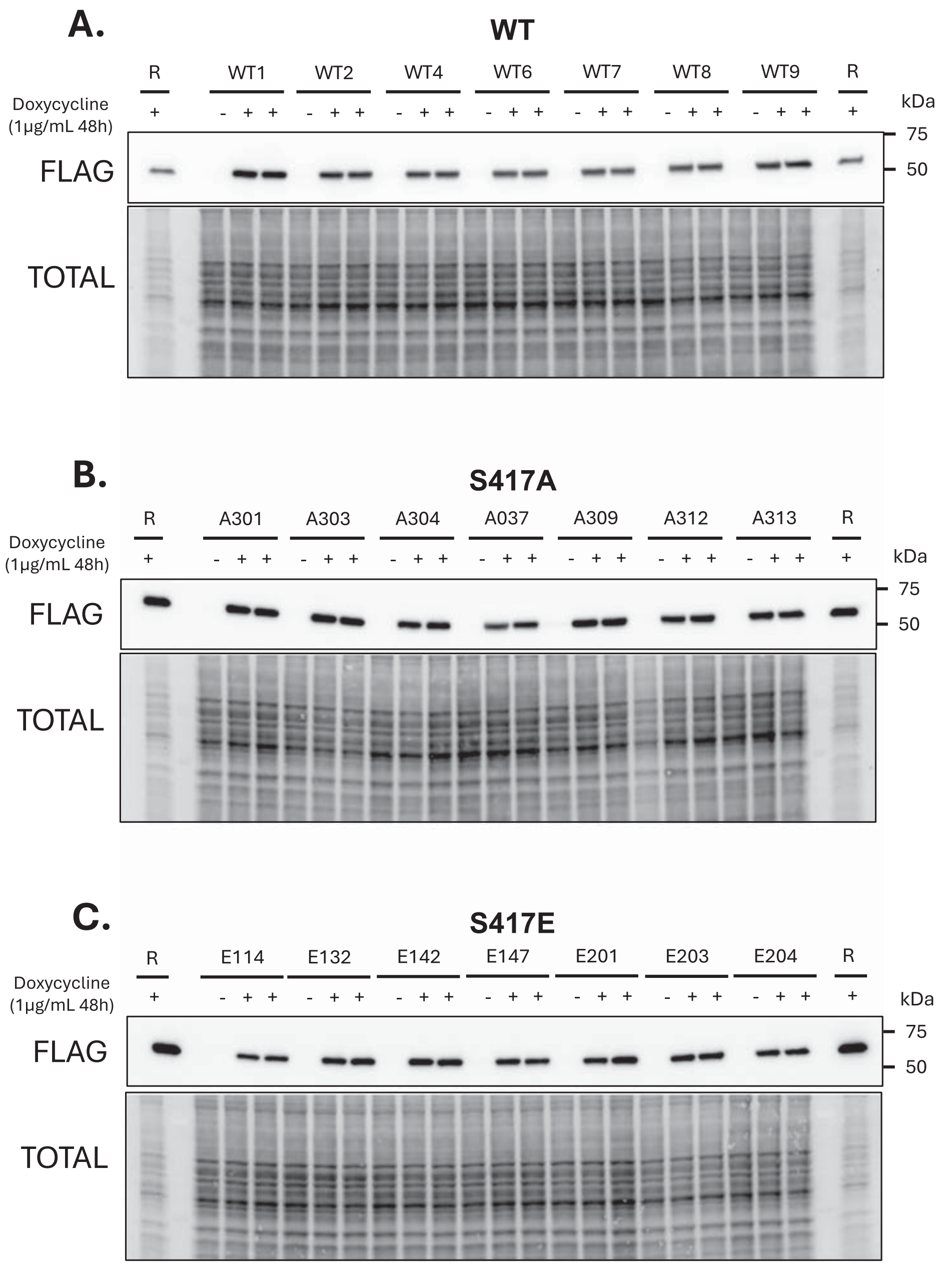

Figure EV5. Representative western blots of the effect of phosphoablative and phosphomimetic mutations in SMAD2.

Individual clones (coded with the indicated numbering) stably transfected with WT (**A**), phosphoablative (**B**) and phosphomimetic (**C**) SMAD2 constructs were non-induced (−) or induced with doxycycline for 48 h. A reference clone (R) induced for 48 h was included for comparison among different western blots. Protein expression was detected with an anti-FLAG antibody. Signals were normalized on the corresponding total protein lanes.

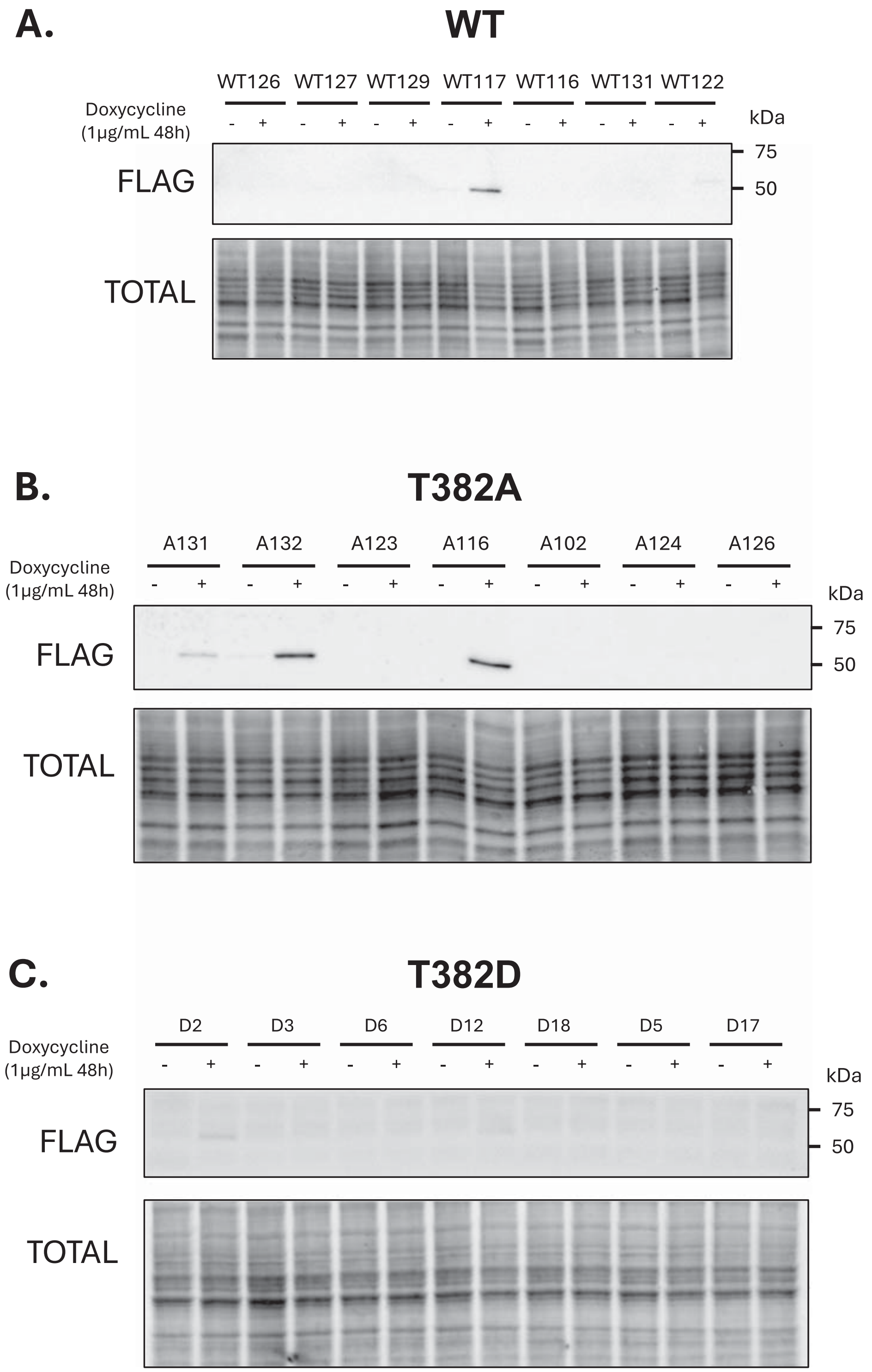

Figure EV6. Representative western blots of the effect of phosphoablative and phosphomimetic mutations in CHK1.

Individual clones (coded with the indicated numbering) stably transfected with WT (**A**), phosphoablative (**B**) and phosphomimetic (**C**) CHK1 constructs were non-induced (−) or induced with doxycycline for 48 h. Protein expression was detected with an anti-FLAG antibody. Signals were normalized on the corresponding total protein lanes.

© The Author(s)

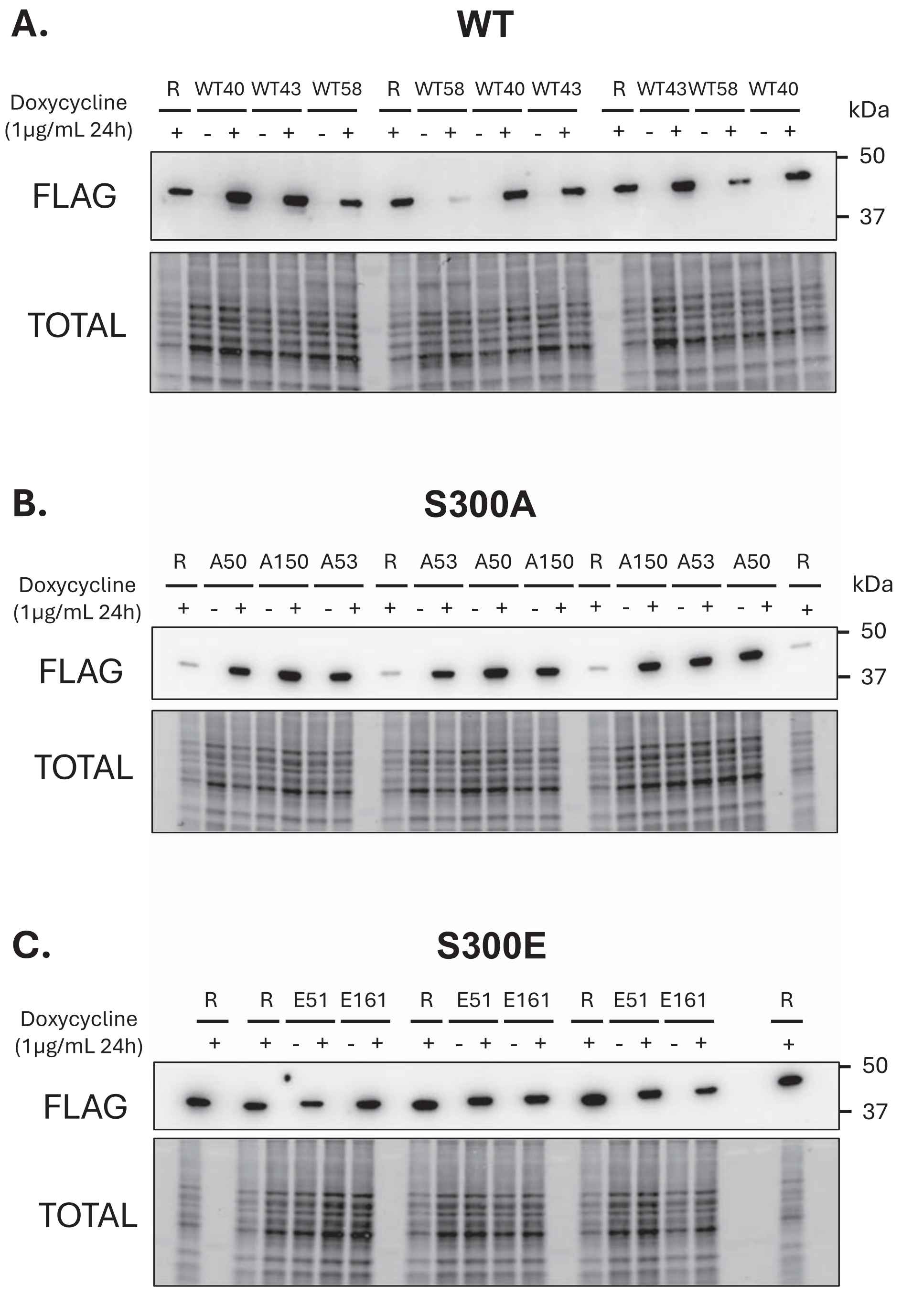

Figure EV7. Representative western blots of the effect of phosphoablative and phosphomimetic mutations in Pyst1.

Individual clones (coded with the indicated numbering) stably transfected with WT (**A**), phosphoablative (**B**) and phosphomimetic (**C**) Pyst1 constructs were non-induced (−) or induced with doxycycline for 24 h. A reference clone (R) induced for 24 h was included for comparison among different western blots. Protein expression was detected with an anti-FLAG antibody. Signals were normalized on the corresponding total protein lanes.

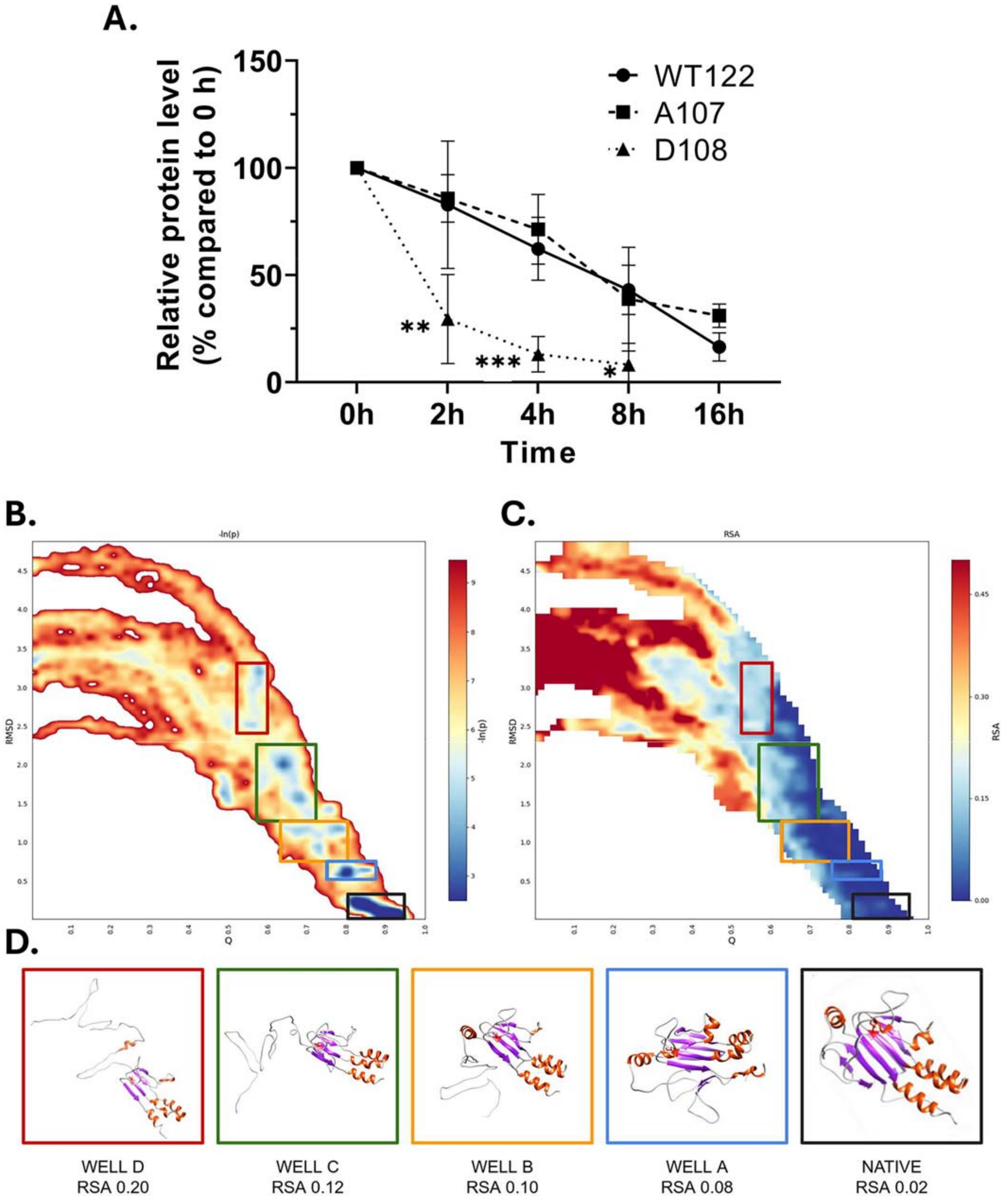
A.
Relative protein level (% compared to 0 h)
150
100
50
0
WT122
A107
D108
**
***
*
0h
2h
4h
8h
16h
Time
B.
-ln(p)
RMSD
Q
C.
RSA
RMSD
Q
D.
WELL D
RSA 0.20
WELL C
RSA 0.12
WELL B
RSA 0.10
WELL A
RSA 0.08
NATIVE
RSA 0.02

 © The Author(s)

Figure EV8. Experimental and computational investigation of cryptic phosphosites in CHK1 and SMAD2.

(**A**) The analysis of CHK1 stability presented in Fig. 5A–C are plotted together to better represent the statistical differences between the phosphomimetic mutant D382 and the WT T382 and phosphoablative mutant A382. The graph illustrates the quantified data (mean ± standard deviation), normalized to the t_0 level. The degradation kinetics were modeled using a one-phase decay curve from which half-time ($t_{1/2}$) values were derived. Data analysis and curve fitting were performed using GraphPad Prism software. Statistical differences vs the WT T382 were obtained by ordinary one-way ANOVA test; **P* < 0.05; ***P* < 0.01; ****P* < 0.001. (**B**) The plot shows the atomistic reconstruction of the SMAD2 folding pathway. Lower-bound approximation of the transition path energy is related to the folding of SMAD2. The energy is plotted as the negative logarithm of the probability distribution (-ln(p)), which is expressed as a function of the collective variables Q and RMSD (65×65 bin matrix). Gaussian blur was applied. The highly populated native state appears as expected in the bottom-right corner (high Q and low RMSD, black rectangle). The indexed squares define the most-populated partially unfolded regions of interest (-ln(p)). (**C**) The plot shows the distribution of RSA of the S417 residue along the transition path. The highest values are associated with the unfolded state (top left), while in the native state the RSA is consistently below 0.15. (**D**) Representative conformations for each cluster. S417 is displayed in red, α-helices are colored in orange, β-sheets in light purple.

© The Author(s)

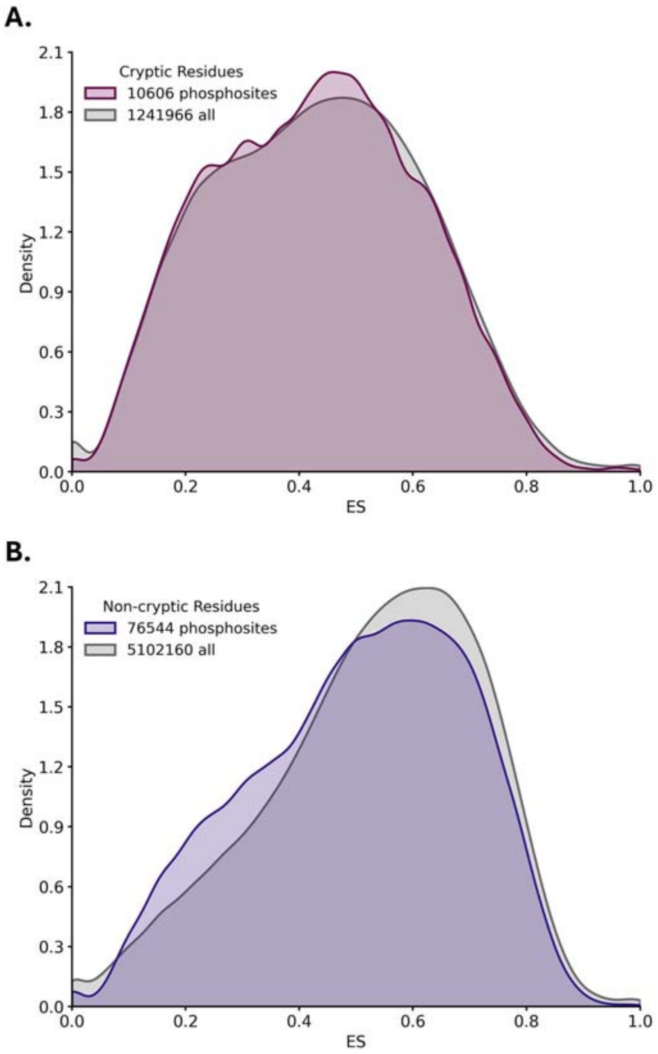

Figure EV9. Graphic distribution of evolutionary conservation of cryptic and non-cryptic phosphosites.

(**A-B**) KDE plot reports the distribution of ES for cryptic (light purple) and non-cryptic (dark purple) phosphosites. The evolutionary conservation of cryptic and non-cryptic phosphosites were superimposed to the distribution of cryptic and non-cryptic amino acids.

© The Author(s)
